# Patterns of Positive Selection of the Myogenic Regulatory Factor Gene Family in Vertebrates

**DOI:** 10.1371/journal.pone.0092873

**Published:** 2014-03-20

**Authors:** Xiao Zhao, Qi Yu, Ling Huang, Qing-Xin Liu

**Affiliations:** Laboratory of Developmental Genetics, Shandong Agricultural University, Tai’an, Shandong, China; University of Minnesota Medical School, United States

## Abstract

The functional divergence of transcriptional factors is critical in the evolution of transcriptional regulation. However, the mechanism of functional divergence among these factors remains unclear. Here, we performed an evolutionary analysis for positive selection in members of the myogenic regulatory factor (MRF) gene family of vertebrates. We selected 153 complete vertebrate MRF nucleotide sequences from our analyses, which revealed substantial evidence of positive selection. Here, we show that sites under positive selection were more frequently detected and identified from the genes encoding the myogenic differentiation factors (*MyoG* and *Myf6*) than the genes encoding myogenic determination factors (*Myf5* and *MyoD*). Additionally, the functional divergence within the myogenic determination factors or differentiation factors was also under positive selection pressure. The positive selection sites were more frequently detected from *MyoG* and *MyoD* than *Myf6* and *Myf5*, respectively. Amino acid residues under positive selection were identified mainly in their transcription activation domains and on the surface of protein three-dimensional structures. These data suggest that the functional gain and divergence of myogenic regulatory factors were driven by distinct positive selection of their transcription activation domains, whereas the function of the DNA binding domains was conserved in evolution. Our study evaluated the mechanism of functional divergence of the transcriptional regulation factors within a family, whereby the functions of their transcription activation domains diverged under positive selection during evolution.

## Introduction

Recent studies in evolutionary genetics have provided several lines of evidence supporting the role of positive selection in the evolution of many genes. These studies have suggested that positive genetic selection is also the major evolutionary force in addition to neutral mutations and random genetic drift [1]–[3]. In all known organisms, transcriptional regulation plays a central role in complex biological processes. However, the mechanisms underlying the functional gain and divergence of transcription factors remain unclear. Here, we performed an evolutionary analysis to study the role of positive selection in the evolution of myogenic regulatory factors (MRFs), which comprise the transcription factor family that regulates myogenesis.

Myogenesis involves two major temporally ordered steps. First, myogenic progenitor cells (myoblasts) originate from mesenchymal precursor cells, and second, these cells then terminally differentiate into mature muscle fibers [4]. The myogenic regulatory factors (MRFs) play key roles in myoblast determination and differentiation [5], [6]. In vertebrates, the MRF family includes myogenic differentiation 1 (*MyoD*), myogenic factor 5 (*Myf5*), myogenin (*MyoG*), and *Myf6* (*MRF4*) genes. All MRFs share a conserved basic helix-loop-helix (bHLH) domain that is required for DNA binding and dimerization with other proteins, such as E protein. All four MRFs are characterized by their capacity to convert a variety of cell lines into myocytes and to activate muscle-specific gene expression [7], [8]. The four MRF proteins display distinct regulatory roles in muscle development. *Myf5* and *MyoD* are myogenic determination factors and contribute to myoblast determination, which is activated in proliferating myoblasts before overt differentiation. In contrast, *MyoG* and *Myf6* are myogenic differentiation factors that contribute to the differentiation of myoblasts and act downstream of *Myf5* and *MyoD*, though *Myf6* partly acts at both the determination and differentiation levels [6], [9], [10]. Although *Myf5* and *MyoD* have redundant functions in myoblast determination and can compensate for the functional loss of each other, *Myf5* plays a more critical role during the early determination of epaxial muscle, whereas *MyoD* is more critical for hypaxial muscle determination [8], [11].

Genome duplication is believed to be a major genetic event that occurs during the evolution of a gene family from a single gene to multiple gene copies [12], [13]. Indeed, evolutionary analyses of the amino acid sequences of the MRF family indicate that vertebrate *Myf5*, *MyoD*, *MyoG*, and *Myf6* genes were duplicated from a single invertebrate gene [14], [15]. The vertebrate genome contains all four MRFs genes, whereas the invertebrate genomes of *Caenorhabditis elegans* [16], *Anthocidaris crassispina* [17], and *Drosophila melanogaster* [18] only contain a single MRF gene. However, although only a single MRF gene exists in the genome of *Ciona intestinalis*, it gives rise two different transcripts of MRFs (*MDFa* and *MDFb*) as a result of alternative splicing. Moreover, in cephalochordates, the amphioxus have two MRF genes: *BMD1* and *BMD2* [19], [20]. The amphioxus and ascidians are chordates species and are closely related to vertebrates [21]. The two MRF genes in amphioxus might be the adaptive result of muscle evolution in cephalochordates in order to acquire a more complex transcriptional regulatory network for myogenesis [15], [22], [23]. The two splice forms of *MyoD* in ascidians suggest that the regulation pattern of multiple *MyoD* genes has evolved under selective pressure before the MRF genes were duplicated into multiple copies [19], [24]. Genome evolution studies suggested that large-scale genome duplications occurred during early chordate evolution [12], [25]. The vertebrate genome appears to undergo two rounds of duplication according to the “one-two-four” rule [13], and the MRF gene family appears to have followed that rule as well [14]. The single ancestral gene initially duplicated into two lineages during the evolution of chordates. The *Myf5* and *MyoD* genes were then duplicated from one of these two lineages, whereas *MyoG* and *Myf6* were duplicated from the other lineage during vertebrate evolution. Therefore, the functional redundancy between *Myf5* and *MyoD* as well as between *MyoG* and *Myf6* might be due to their common genetic origin [14].

The mechanisms underlying the evolution of the MRF gene family during their duplication remain unclear. In particular, the evolutionary forces affecting the functional divergence of the four MRFs genes have not been fully elucidated. In this study, we investigated the mechanisms underlying the evolution of the four MRF genes with particular emphasis on the selective pressures imposed on the branches and sites of MRFs during vertebrate evolution. Our study provides several lines of evidence for the role of positive selection in the functional divergence of transcription factors.

## Results

### The sequence variations among the four groups of vertebrate MRFs

**Figure 1:**
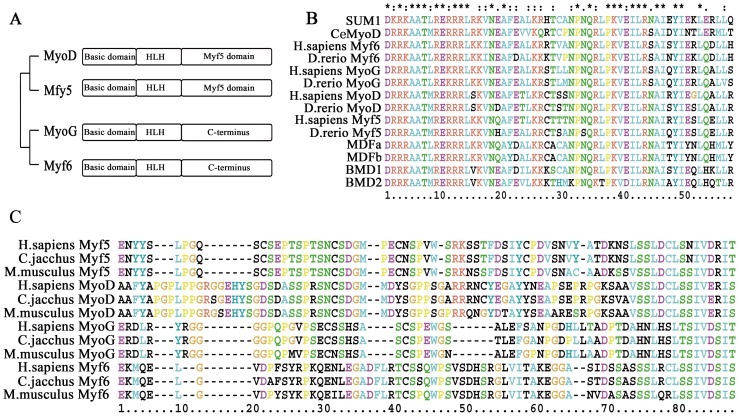
The protein sequence alignment of the MRF family. **A**) The domain differences of the MRFs gene family. **B**) The sequence alignment of the HLH domains of representative MRFs from nematodes to humans. **C**) The sequence alignment of the C-terminal sequences of representative vertebrate MRFs. The amino acid sequence SXXTSPXSNCSDGM and SSLDCLSXIVXRIT are conserved in the MYF5 domains of *MyoD* and *Myf5*.

In vertebrates, the MRF sequences were divided into four groups and their protein structure differences are shown in Figure 1A. Three functional domains were identified in MRF proteins by querying the Conserved Domain Database in NCBI [26]. The most conserved region is the HLH domain, which defines the MRF family, as its amino acid sequences were almost unchanged among the four MRFs (Fig. 1A and 1B). The BASIC domain was also conserved in all of the MRFs (Fig. 1A). However, the third MYF5 domain was only conserved in the myogenic determination factors (*Myf5* and *MyoD*), but not in the myogenic differentiation factors (*Myf6* and *MyoG*) (Fig. 1A). Two amino acid sequences of SXXTSPXSNCSDGM and SSLDCLSXIVXRIT were highly conserved in the MYF5 domain of *Myf5* and *MyoD* (Fig. 1C).

### Detection of positive genetic selection for all vertebrate MRFs sequences

**Figure 2:**
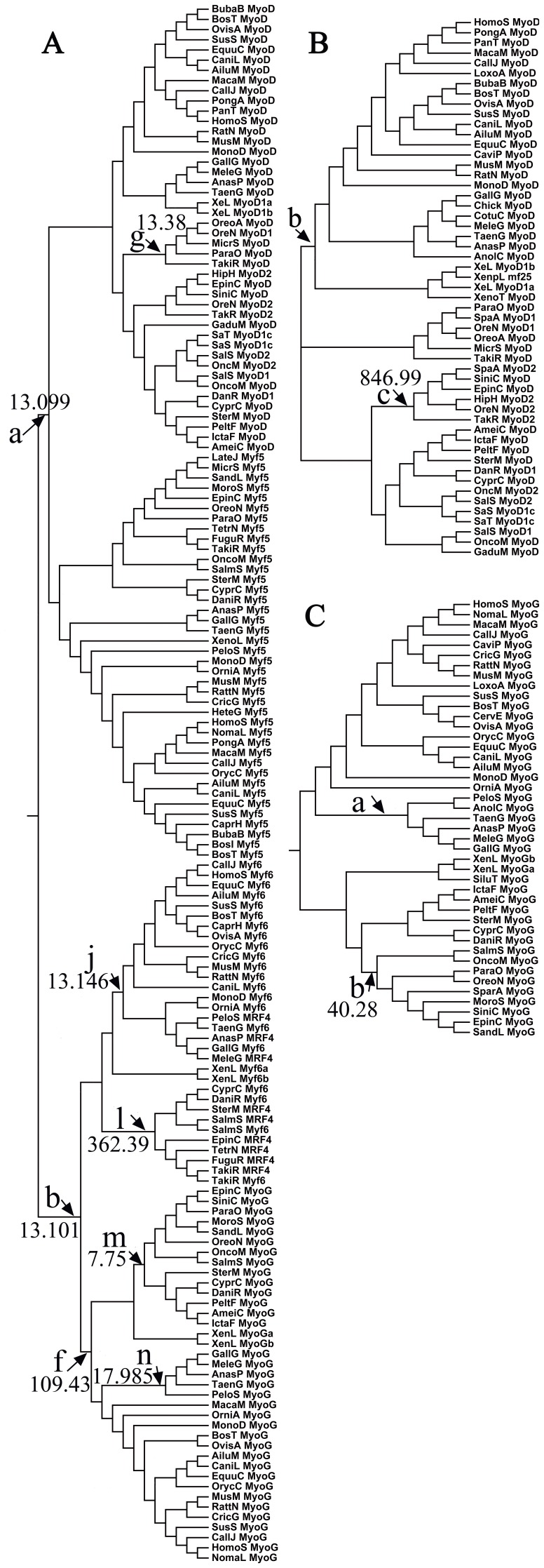
Estimation of positive selection during MRFs evolution. The branches were estimated for positive selection in the following: **A**) vertebrate MRFs phylogeny, **B**) vertebrate *MyoD*; and, **C**) vertebrate *MyoG*. All the branches with a ω-ratio significantly greater than 1 are marked with arrows and letters corresponding to those in Table 1 and Table 2.

**Figure 3:**
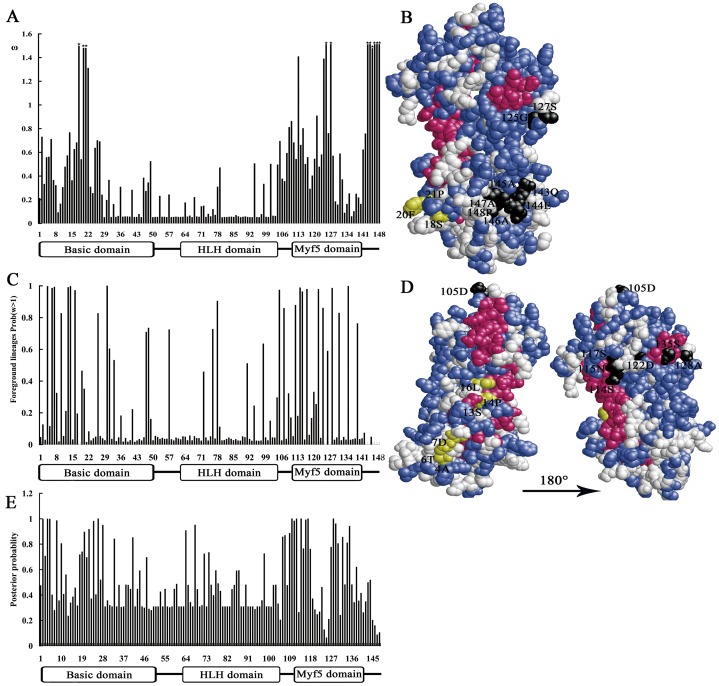
Mapping positive selection sites for the functional divergence between myogenic determination factors and myogenic differentiation factors. **A**, **B**) Maps of the positive selection sites identified using all vertebrate MRF sequences. The stars represent the 11 sites under positive selection identified by M8 versus M7 in Table 1. **C**, **D**) The map sites under positive selection responsible for the functional divergence between myogenic determination factors and myogenic differentiation factors. The sites with Bayes Empirical Bayes (BEB) probabilities>0.95 represent the sites under positive selection in Table 1. The yellow balls represent the sites located in the BASIC domain, and black balls represent the sites located in the MYF5 domain and C-terminus. The position of positive selection sites on the protein three-dimensional MRFs model are marked according to the sequences of human *MyoD*. **E**) Twenty-nine residues with a posterior ratio more than 8 have been observed as Type I functional divergence.

Nucleotide mutations in coding sequences are important for the evolution of gene functions. The likelihood ratio (LR) tests of site models in the CODEML program of phylogenetic inference by maximum likelihood (PAML4) [27] were used to test the positive selection of all vertebrate MRF sequences. A neighbor joining (NJ) tree of 153 vertebrate MRF coding sequences (File S1) was used for the LR tests (Fig. 2A). The LR tests with M7 and M8 detected positive selection by using all vertebrate MRFs sequences, which fit the selective model better than the null model and also had a ω>1 (Table S1). The results remain significant with the experimental error set at 1% (Table 1, Table S1). There are 11 sites under positive selective pressure, which were identified under M8 using Bayes Empirical Bayes (BEB) analysis [28], [29] (Table 1, Table S1, Figs. 3A and 3B).

**Table 1:** Likelihood Ratio Tests for the Positive Selection on all the MRF genes.

Lineages	Model		Parameters	Positively Selected Sites		Null	Positive		2Δ	
Vertebrates	Site model									
	M8 vs M7		ω = 2.4396,p = 0.00001	18S*,20F*,21P*,125G**,127S**,143Q**,144E**,145A*,146A**,147A**,148P**	−20606.91			−25259.2		8695**
	Branch-site model									
	Ha vs Ha0		ω = 13.099, p = 0.236	4A**,6T*,7D**,13S**,14P**,16L*, 30Q**, 105D*, 114S*, 115N*, 117S*, 122D*,128S*, 135S**	−21226.78			−21222.8		7.89**
	Hb vs Hb0		ω = 13.101, p = 0.236	4A**,6T*,7D**,13S**,14P**,16L*, 30Q**, 105D*, 114S*, 115N*, 117S*, 122D*, 128S*, 135S**	−21226.78			−21222.8		7.89**
	Hf vs Hf0		ω = 109.43, p = 0.162	25V*,79S*,118D**,120M*,124A*	−21233.63			−21231.7		4*
	Hg vs Hg0		ω = 13.39, p = 0.0486	20F**, 22A**, 126K*	−21235.8			−21232.4		7**
	Hj vs Hj0		ω = 13.146, p = 0.0383	109Y*, 113R*	−21236.95			−21234.5		5*
	Hl vs Hl0		ω = 27.007, p = 0.067	31A*, 111A**	−21235.2			−21232.8		4.87*
	Hm vs Hm0		ω = 7.7495, p = 0.0952	112P**, 116C**, 128S**	−21233.42			−21230.9		5.01**
	Hn vs Hn0		ω = 17.985, p = 0.063	27A*, 101A**	−21236.42			−21234.4		4*
	Branch model									
	M0 vs Free- ratio-model	ωb = 568.98,ωc = 494.43,ωe = 541.95,ωh = 1.027, ωi = 223.93,ωk = 468.83,ωl = 362.39,ωo = 507.55			−21408.74			−21096.3		624**
	M0 vs Two- ratio-model	ω0 = 0.055, ωc = 999.00			−21408.74			−21406.5		4.6*
		ω0 = 0.055, ωd = 999.00			−21408.74			−21404.8		8**

The ω represents for Ka/Ks, the topology and branch-specific ω ratios are presented in Figure 3. * Significant at p<0.05, ** Significant at p<0.01. The site number is marked with the alignments with the gap eliminated. 2Δ, log-likelihood difference between compared models.

### Different positive selection on the four branches of vertebrate MRFs

Typically, the relatively short period of positive selection is usually followed by long periods of continuous negative selection [2]. The branch models of the CODEML program were used to examine whether some branches in the MRFs phylogeny were driven by positive selection. First, we used the one-ratio model (M0), which assumes a single ω ratio for all lineages in the phylogeny [28], [30]. Under the M0 model, the ω ratio is 0.055, which is significantly less than 1, and indicates that the evolution of MRFs was dominated by strong purifying selection (Table 1). We then used free-ratio and two-ratio models to test for positive selection in each branch. The free-ratio model assumes a different ω parameter for each branch in the tree [28], [30]. The LR test results revealed that the differences between the free-ratio and one-ratio models were significant (p<0.01, Table 1), indicating that the ω ratios were different among the lineages.

**Figure 4:**
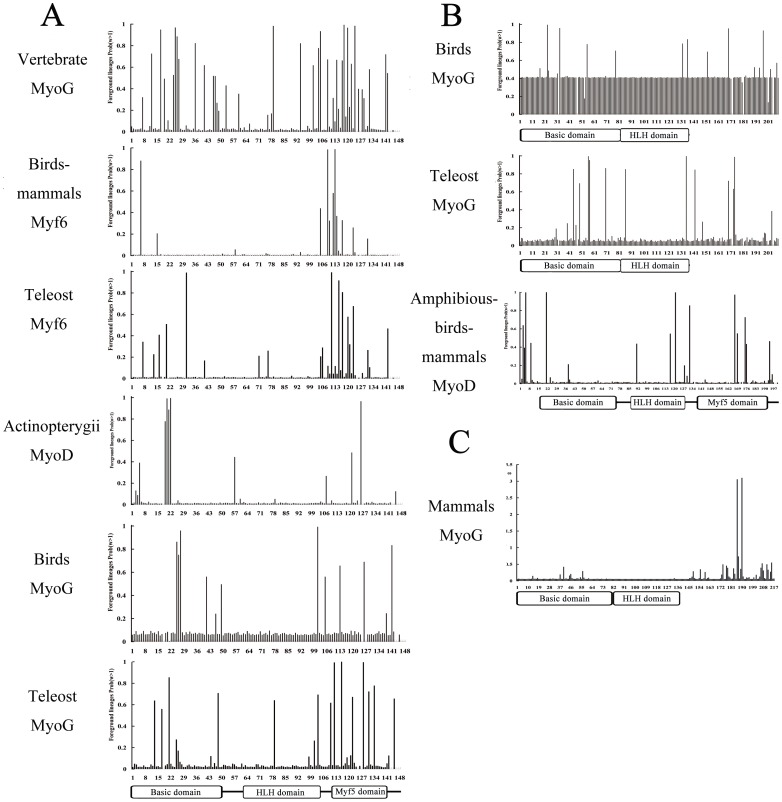
Mapping positive selection sites for the functional divergence among members of MRFs. **A**) Positive selection sites identified from lineages of vertebrate MRFs. **B**) Positive selection sites identified from lineages of vertebrate *MyoD* or *MyoG*. **C**) Positive selection sites identified from the mammalian *MyoG* sequences. The sites with Bayes Empirical Bayes (BEB) probabilities >0.95 represent the sites under positive selection in Table 2.

Given that positive selection usually affects a few amino acid sites along particular lineages [2], [27], we used branch-site models to further examine whether some sites along particular MRFs lineages are under positive selection pressure (Table 1). As expected, the positive selection on the four vertebrate MRF lineages was different. We identified 5 sites under positive selection from the vertebrate *MyoG* lineage (branch f in Fig. 2A, Fig. 4A and Table 1). In addition, another 3 and 2 amino acid sites were identified from the teleost *MyoG* lineage and the bird *MyoG* lineage, respectively (branches m and n in Fig. 2A, Fig. 4A, and Table 1). Although no positive selection sites were identified in the entire vertebrate *Myf6* lineage, 2 sites were identified from the birds-mammals *Myf6* lineage, and 2 additional sites were identified in the teleost *Myf6* lineage (branches j and l in Fig. 2A, Fig. 4A, and Table 1). In addition, only 3 sites were identified from the Actinopterygii *MyoD* lineage (branch g in Fig. 2A, Fig. 4A, and Table 1). However, no site was identified from the *Myf5* lineage.

### The functional divergence between the myogenic determination factors (*Myf5/MyoD*) and myogenic differentiation factors (*MyoG/Myf6*)

The myogenic determination factors (*Myf5/MyoD*) and myogenic differentiation factors (*MyoG/Myf6*) play distinct roles in myogenesis. The functional divergence between these factors was estimated using the DIVERGE 2.0 program [31]. Type I functional divergence showed θ = 0.499±0.04 between *Myf5/MyoD* and *MyoG/Myf6* branches, which was significantly greater than 0 (p<0.01). Thus, the functional divergence between *Myf5/MyoD* and *MyoG/Myf6* was significant. Twenty-nine residues have a stringent threshold of a posterior ratio higher than eight. Most of these sites were located in the BASIC, MYF5 domains and C-terminus, which might be critical for the functional divergence between the myogenic determination factors (*Myf5* and *MyoD*) and myogenic differentiation factors (*Myf6* and *MyoG*) (Fig. 3E). The role of positive selection in this divergent process was evident (Table 1, Fig. 2A, and Fig. 3C). Using the branch-site specific model, the same 14 positive selection sites were identified from the *Myf5/MyoD* lineage (branch a in Fig. 2A) and *MyoG/Myf6* lineage (branch b in Fig. 2A), with 7 of them located in the BASIC domain, 1 close to the HLH domain, and 6 in the MYF5 domain and C-terminus (Fig. 3C, Fig. 3D, and Table 1).

### Detection of positive genetic selection for each group of vertebrate MRF sequences

A neighbor joining (NJ) tree of 53 vertebrate *MyoD* coding sequences (File S2) was generated, which was used for positive selection analysis (Fig. 2B). No sites were identified using the site models. However, positive selection was identified from the teleost *MyoD2* lineage using the two ratio branch model (branch c in Fig. 2B, Table 2). Moreover, 4 sites were identified from the lineage of the amphibians-birds-mammals *MyoD* (branch b in Fig. 2B, Fig. 4B and Table 2). Thus, the evolution of *MyoD* for all vertebrates was likely driven by positive selection. Similarly, positive selection was identified in vertebrate *MyoG* using a tree of 43 sequences (Fig. 2C and File S3). Using a branch-site model, 3 sites were identified from the lineage of the bird *MyoG* (branch a in Fig. 2C, Fig. 4B and Table 2) and 4 sites were identified from the teleost *MyoG* lineage (branch b in Fig. 2C, Fig. 4B and Table 2). Unlike the other MRF genes, 2 sites were still identified by the pair model of M7 versus M8 when the sequences were limited only to the 19 mammalian *MyoG* sequences (Table 2, Fig. 4C and File S4). These results suggest that the evolution of *MyoG* in all vertebrates was driven by positive selection.

**Table 2:** Likelihood Ratio Tests for the Positive Selection on each of the four MRFs.

Lineage	Model	Parameters	Positive Selection Sites	Null	Positive	2Δ
Vertebrate MyoD	Branch model					
	M0 vs Free-ratio model	ωa = 999.00, ωb = 2.97, ωc = 999.00	none	−9667.47	−9528.87	277.2**
	M0 vs two-ratio model	ω0 = 0.054, ωc = 846.99	none	−9667.47	−9665.47	4*
	Branch-site model					
	Hb vs Hb0	ω = 999.00, p = 0.054	5C** 21P** 121G** 167A*	−9574.6	−9567.28	14.64**
Vertebrate MyoG	Branch-site model					
	Ha vs Ha0	ω = 999.00, p = 0.06	23P** 33G* 169A*	−9170.65	−9166.27	8.76**
	Hb vs Hb0	ω = 40.28, p = 0.051	56P** 57E* 135S** 174 N*	−9170.1	−9165.24	9.6**
Mammal MyoG	Site model					
	M8 vs M7	p = 0.009, ω = 3.04	187T* 191T**	−3805.78	−3795.83	19.9**
Vertebrate Myf6	Branch model					
	M0 vs free-ration model	ωa = 999.00	none	−6577.13	−6491.56	171.2**

The ω represents for Ka/Ks, the topology and branch-specific ω ratios are presented in Figure 3. *Significant at p<0.05, ** Significant at p<0.01. The site number is marked with the alignments with the gap eliminated. 2Δ, log-likelihood difference between compared models.

Unlike *MyoD* and *MyoG*, no branch or site under positive selection was identified in the vertebrate *Myf5* gene (Table S2). Although the selective pressures on the branches of *Myf6* were different (Table 2), no sequences were found to be under positive selection at the 5% confidence level (Table S2). These data suggest that the evolution of *MyoD* and *MyoG* was driven strongly by positive selection, but the evolution of *Myf5* and *Myf6* was only weakly driven by this selective pressure.

### Location of positive selection sites

Under Bayes Empirical Bayes (BEB) analysis, a total of 55 positive selection sites during the divergence of MRFs were identified using the site and branch-site models of PAML4. We plotted the genetic location of positively selected sites onto the protein secondary structure and three-dimensional structure (Fig. 3 and Fig. 4). Positively selected sites were not homogeneously distributed among regions. A total of 40% (22 of 55) of sites were located in the BASIC domain, whereas 51% (28 of 55) of sites were located in the MYF5 domain and C-terminus. Only 2 sites were located in the HLH domain. Among the 28 sites in the MYF5 domain, most were located in conserved amino acid sequences of SXXTSPXSNCSDGM and SSLDCLSXIVXRIT. To identify connections between positive selection and functional sites, spatial relationships among the positive selection sites were evaluated by mapping them onto three-dimensional protein structures [32], [33]. All sites were shown to localize on the protein surface (Fig. 3B and 3D).

### Different rates of evolution for each of the three MRFs domains

**Figure 5:**
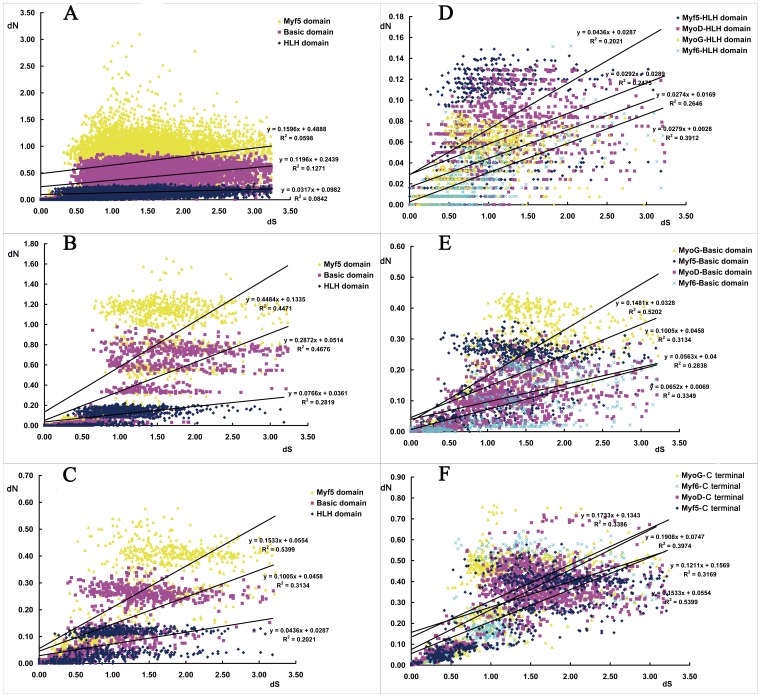
Nonsynonymous substitution rate (dN) and synonymous substitution rate (dS) of the three domains in the MRFs. **A**), **B**) and **C**) represent the dN/dS differences of the three domains of the MRFs in vertebrates, mammals and *Myf5* genes, respectively. **D**), **E**) and **F**) represent the dN/dS differences of the four MRFs genes in their HLH, BASIC and MYF5 domains, respectively.

Given that most of the positive selection sites are frequently located in the BASIC and MYF5 domains of MRF proteins, the positive selection pressures on the three domains should be different. Thus, the evolution rates of the three domains were analyzed by calculating the nonsynonymous (*dN*) and synonymous (*dS*) substitution rates (Fig. 5). The MYF5 domain had the fastest evolutionary rate, whereas the HLH domain evolved the slowest (Fig. 5A, 5B, and 5C). In addition, the evolutionary rate of C-terminal sequences in *MyoG* and *Myf6* was significantly faster than the MYF5 domain of *MyoD* and *Myf5*, whereas the HLH domain had a similar evolutionary rate among the four MRFs (Fig. 5D and 5F).

## Discussion

The four MRF genes display distinct regulatory roles during embryonic myogenesis and postnatal muscle development [6], [9], [34]. However, the mechanisms underlying the functional divergence among them remain unclear. In this study we investigated the evolution of the four MRF genes in order to determine the role of positive selection in the functional divergence of this transcription factor family.

### The functional complex trajectories of vertebrate MRFs genes

The four vertebrate MRF genes diverged from a single invertebrate ancestor gene following two rounds of genomic duplication [14]. In the urochordate *Ciona intestinalis*, two MRF proteins (MDFa and MDFb) were transcribed by a single MRF gene, which was different than lower invertebrates, whereby a single MRF ortholog was transcribed [35]. Thus, the vertebrate-like regulatory strategy of multiple myogenic factors has been described in *Ciona intestinalis* [19], [24], [35]. In vertebrates, the four MRFs are produced by gene duplication. It has been shown that *Myf5* and *MyoD* evolved from one of these lineages, whereas *MyoG* and *Myf6* (*MRF4*) evolved from another lineage [14], which might explain the functional overlap of these factors [6], [7]. All three domains of MRF proteins were identified in vertebrates. The HLH and BASIC domains were conserved in all of the vertebrate MRFs. However, the third MYF5 domains were only identified in the vertebrate Myf5 and MyoD genes, but are not conserved in *Myf6* and *MyoG* (Fig. 1A). Therefore, the MYF5 domain is critically involved in the functional differences between the myogenic determination factors (*Myf5* and *MyoD*) and the myogenic differentiation factors (*MyoG* and *Myf6*). In addition, two amino acid regions (SXXTSPXSNCSDGM and SSLDCLSXIVXRIT) might be critical in the functional gain of the myogenic determination role in *Myf5* and *MyoD* (Fig. 1C). Most sites of the SSLDCLSXIVXRIT region were also conserved in the *Myf6* C-terminus, which might explain the minor role of *Myf6* in myogenic determination (Fig. 1C) [6], [10].

### The functional divergence between the myogenic determination factors (*Myf5/MyoD*) and myogenic differentiation factors (*MyoG/Myf6*)

Positive selection and gene duplication are two major forces in the adaptive evolution of new functions in a gene family [2]. Significant evidence of positive selection was found during the evolution of the vertebrate MRFs. Positively selected sites were identified in the BASIC, MYF5 domains and C-terminus, and all of these sites localized on the surface of human *MyoD* (Fig. 3A and 3B). Given that the BASIC, MYF5 domain and C-terminus are the transcription activation domains and are required for muscle gene activation [6], [7], the positive selective pressures may alter the capability of MRFs to activate myogenic gene expression, which might be responsible for the functional divergence of the vertebrate MRFs.

Indeed, our findings provide evidence that the functional divergence of the transcriptional activity domain between the myogenic determination factors (*Myf5* and *MyoD*) and differentiation factors (*Myf6* and *MyoG*) was driven by positive selection. Positive selection sites responsible for this divergent process were identified from the BASIC, MYF5 domains and C-terminus (Fig. 2A, Fig. 3C and 3D). Moreover, the role of positive selection in functional divergence between *Myf5/MyoD* and *MyoG/Myf6* was also evident after examining the selective pressure on each of the four vertebrate MRFs lineages, which suggested that the major sites and species under positive selection were observed in the *MyoG* and *Myf6* lineages, while few were identified in *Myf5* and *MyoD* (Fig. 2A and Fig. 4A). In particular, positive selection sites in the HLH domain were identified from the vertebrate *MyoG* branch (Table 1, Fig. 2A and Fig. 4A). The HLH domain is required for DNA binding and dimerization of myogenic bHLH factors with other proteins [7], [10]. Thus, the transcriptional activity domain and DNA binding domain of *MyoG* were all likely driven by positive selection pressures, which could explain the specific role of *MyoG* in myogenic differentiation, but not in myogenic determination [36]. Although sites located in the C-terminus were also identified from two *Myf6* branches in a number of organisms ranging from teleosts to mammals, no sites were located in the conserved regions (Fig. 2A, Fig. 4A, and Table 1). This may explain the more specific role of *Myf6* in both myogenic differentiation and myogenic determination [10], [36], [37]. Conversely, only a few sites in the *Myf5* and *MyoD* lineages were identified, suggesting that the functions of myogenic determination factors were more conserved during their divergence from the ancestral gene. Overall, the myogenic differentiation factors gained new functions under positive selective pressure, while myogenic determination factors mostly retained the basic functions of ancestral bHLH genes. These observations could explain the more important and conserved functions of *MyoD/Myf5* than *Myf6/MyoG* in the regulation of muscle development [10], [36].

### The functional divergence between the myogenic determination factors *Myf5* and *MyoD*

In addition to the divergence between the myogenic determination factors and differentiation factors, the functional divergence within the myogenic determination factors (between *Myf5* and *MyoD*) was also under positive selective pressure. The evolution processes of *MyoD* in all vertebrates are driven by positive selection on the BASIC and MYF5 domains (Fig. 2B, Fig. 4B, and Table 2). However, no branches or sites under positive selection were identified during *Myf5* evolution, which was selected by purifying selection. The different positive selective pressure between *Myf5* and *MyoD* might explain the functional divergence between myogenic determination factors because *MyoD* gained new functions during its evolution from amphibians to mammals [38]–[40], whereas *Myf5* functions remained conserved after its divergence.

### The functional divergence between the myogenic differentiation factors *MyoG* and *Myf6*

Similar to the myogenic determination factors, the function of myogenic differentiation factors (*Myf6* and *MyoG*) also diverged under positive selection. The positive selection on the BASIC and C-terminus were identified in the bird *MyoG* lineage and the teleost *MyoG* lineage (Fig. 4B and Table 2). In addition, unlike other MRF genes, positive selection was identified, though the estimate was limited to the mammalian *MyoG* sequences (Table 2 and Fig. 4C). Thus, the evolution of *MyoG* in all vertebrates was under positive selection. However, positive selection was not identified during *Myf6* evolution, which indicated a relatively slow evolution rate of *Myf6* after its divergence from myogenic differentiation factors. Therefore, although *Myf6* and *MyoG* were duplicated from the same ancestral gene, the functions of *Myf6* are different from *MyoG* [6], [9], [10].

### The different positive selection of the three vertebrate MRFs domains

The HLH domain is crucial for the MRF family, and therefore its amino acid sequences are almost unchanged during the evolution from nematodes to humans. In contrast, the sequences of the BASIC, MYF5 domains and C-terminus show a greater number of differences among species (Fig. 1A and Fig. 1B). Indeed, positive selection sites were identified in the BASIC, MYF5 domains and C-terminus, whereas few were found in the HLH domain. Therefore, the role of the three domains in the evolution and functional divergence of the MRF genes might be different. Based on evolutionary analysis, the role of the HLH domain in maintaining the conserved function of the MRF gene family was confirmed, whereas the BASIC, MYF5 domains and C-terminus are the targets for the gain of new functions under positive selective pressure. Thus, the DNA binding features among the four MRF genes are similar due to the conserved HLH domain. However, the transcriptional activity features among them vary due to the different evolutionary rates of the BASIC, MYF5 domains and C-terminus. Thus, their transcriptional activity for specific muscle genes are different, which resulted in their distinct roles in myogenesis [6], [36], [41].

Overall, we conclude that the functional gain and divergence of these transcription factors were driven by distinct positive selection on their transcription activation domains, whereas the DNA binding domains play roles in maintaining the conserved function of the transcription factor family.

## Materials and Methods

### Data collection and alignment

BLASTP, TBLASTN and keyword searches were used to obtain the open reading frames of MRFs from the NCBI (http://www.ncbi.nlm.nih.gov/guide/). The MRF sequences were aligned by the program MUSCLE or ClustalW, and all gaps were eliminated by manual edition (File S1). The alignment results were used to calculate the selection pressure with PAML4 [27]. The MRF protein structures were mapped by querying the Conserved Domain Database in NCBI [26].

### Phylogenetic analyses

Phylogenetic trees were constructed using the MEGA5 software [42] with the Neighbor-Joining (NJ) method, a mathematical model of P-distance, 1000 bootstrap replicates, and complete deletion. In addition, the maximum likelihood (ML) trees for the MRFs were also constructed with the MEGA5 software using Kimura-2 parameters, 1000 bootstrap replicates, and complete deletion.

### Detection of the evolutionary rates for MRF coding sequences

The CODEML program in the PAML4 [27] was used to calculate the positive selection of the MRFs. In the CODEML program, the branch model allows the ω ratio to vary among branches in the phylogeny [28], [43]. In branch models, the simplest model is M0, which is referred to as the null hypothesis H0, and it assumes the same ω ratio for all branches. The model = 1 fits the free-ratio model, which assumes an independent ω ratio for each branch. The model = 2 fits the two-ratio model, which is allowed to have several ω ratios [27]. The site model allows the ω ratio to vary among sites (amino acids in the protein). In the site model analysis, two pairs of models appeared to be particularly useful, and formed likelihood ratio tests of positive selection. The first compares M1a (Nearly Neutral) and M2a (Positive Selection), whereas the second compares M7 (beta) and M8 (beta and ω). M1a allows two classes of ω sites: negative sites with ω0<1 and neutral sites with ω1 = 1, whereas M2a adds a third class with ω2 possibly >1. M7 allows ten classes of ω sites between 0 and 1 according to a beta distribution with parameters p and q, whereas M8 adds an additional class with ω possibly >1, similar to M2a. In addition, to test whether variable selection pressures exist among the MRFs sites, we also used a paired model of M0 (one-ratio) against M3 (discrete). M3 specifies 3 discrete classes of MRFs coding sites. The branch-site models allows ω ratio to vary in sites and branches on the tree, and used to detect positive selection that affects a few sites along particular lineages (called foreground branches). The nonsynonymous (*dN*) and synonymous (*dS*) substitution rates were calculated by the Nei-Gojobrotri (Jues-Cantor) method as implemented in the MEGA5.0 program to measure the pairwise sequence distances of the three domains among different MRFs [3], [42].

### Three-dimensional structural analyses

Three-dimensional structures of the proteins were predicted using the worldwide web following the methods of a case study using the Phyre server [44]. The structural images for the proteins were produced using RasMol 2.7.5 [45], [46].

### The detection of functional divergence of MRF genes

The DIVERGE 2.0 program [31] was used to estimate the Type I functional divergence between myogenic determination factors (*Myf5/MyoD*) and myogenic differentiation factors (*MyoG/Myf6*). The Type I functional divergence was measured as the coefficient of functional divergence, θ (ranging from 0–1), which was calculated by model-free estimation (MFE) and maximum-likelihood estimation (MLE) under a two-state model. The value of θ represents the functional divergence [47], [48].

## Supporting Information

Table S1 Likelihood Ratio Tests for the positive selection on all MRF genes.
(XLS)

Table S2 Likelihood Ratio Tests for the positive selection on each of the MRFs.
(XLS)

File S1 **Alignment results for the 153 vertebrate MRF coding sequences.**
(NEXUS)

File S2 **Alignment results for the 53 vertebrate** ***MyoD*** **coding sequences.**
(NEXUS)

File S3 **Alignment results for the 43 vertebrate** ***MyoG*** **coding sequences.**
(NEXUS)

File S4 **Alignment results for the 19 mammalian** ***MyoG*** **coding sequences.**
(NEXUS)

## References

[1] Castoe TA, de Koning AP, Kim HM, Gu W, Noonan BP, et al (2009) Evidence for an ancient adaptive episode of convergent molecular evolution. Proc Natl Acad Sci U S A 106: 8986–8991. 19416880 10.1073/pnas.0900233106 PMC2690048

[2] Shen YY, Liang L, Zhu ZH, Zhou WP, Irwin DM, et al (2010) Adaptive evolution of energy metabolism genes and the origin of flight in bats. Proc Natl Acad Sci U S A 107: 8666–8671. 20421465 10.1073/pnas.0912613107 PMC2889356

[3] Jin W, Wu DD, Zhang X, Irwin DM, Zhang YP (2012) Positive Selection on the Gene RNASEL: Correlation between Patterns of Evolution and Function. Mol Biol Evol 29: 3161–3168. 22513284 10.1093/molbev/mss123

[4] Fujisawa-Sehara A (2000) Development and regeneration of skeletal muscle. Tanpakushitsu Kakusan Koso 45: 2228–2234. 11021228

[5] Buckingham M (2001) Skeletal muscle formation in vertebrates. Curr Opin Genet Dev 11: 440–448. 11448631 10.1016/s0959-437x(00)00215-x

[6] Buckingham M, Vincent SD (2009) Distinct and dynamic myogenic populations in the vertebrate embryo. Curr Opin Genet Dev 19: 444–453. 19762225 10.1016/j.gde.2009.08.001

[7] Berkes CA, Tapscott SJ (2005) MyoD and the transcriptional control of myogenesis. Semin Cell Dev Biol 16: 585–595. 16099183 10.1016/j.semcdb.2005.07.006

[8] Parker MH, Seale P, Rudnicki MA (2003) Looking back to the embryo: defining transcriptional networks in adult myogenesis. Nat Rev Genet 4: 497–507. 12838342 10.1038/nrg1109

[9] Bryson-Richardson RJ, Currie PD (2008) The genetics of vertebrate myogenesis. Nat Rev Genet 9: 632–646. 18636072 10.1038/nrg2369

[10] Bentzinger CF, Wang YX, Rudnicki MA (2012) Building muscle: molecular regulation of myogenesis. Cold Spring Harb Perspect Biol 4.. 10.1101/cshperspect.a008342 PMC3281568 22300977

[11] Kablar B, Asakura A, Krastel K, Ying C, May LL, et al (1998) MyoD and Myf-5 define the specification of musculature of distinct embryonic origin. Biochem Cell Biol 76: 1079–1091. 10392718

[12] Sidow A (1996) Gen(om)e duplications in the evolution of early vertebrates. Curr Opin Genet Dev 6: 715–722. 8994842 10.1016/s0959-437x(96)80026-8

[13] Meyer A, Schartl M (1999) Gene and genome duplications in vertebrates: the one-to-four (-to-eight in fish) rule and the evolution of novel gene functions. Curr Opin Cell Biol 11: 699–704. 10600714 10.1016/s0955-0674(99)00039-3

[14] Atchley WR, Fitch WM, Bronner-Fraser M (1994) Molecular evolution of the MyoD family of transcription factors. Proc Natl Acad Sci U S A 91: 11522–11526. 7972095 10.1073/pnas.91.24.11522 PMC45263

[15] Yuan J, Zhang S, Liu Z, Luan Z, Hu G (2003) Cloning and phylogenetic analysis of an amphioxus myogenic bHLH gene AmphiMDF. Biochem Biophys Res Commun 301: 960–967. 12589806 10.1016/s0006-291x(03)00081-0

[16] Krause M, Fire A, Harrison SW, Priess J, Weintraub H (1990) CeMyoD accumulation defines the body wall muscle cell fate during C. elegans embryogenesis. Cell 63: 907–919. 2175254 10.1016/0092-8674(90)90494-y

[17] Venuti JM, Goldberg L, Chakraborty T, Olson EN, Klein WH (1991) A myogenic factor from sea urchin embryos capable of programming muscle differentiation in mammalian cells. Proc Natl Acad Sci U S A 88: 6219–6223. 2068103 10.1073/pnas.88.14.6219 PMC52054

[18] Michelson AM, Abmayr SM, Bate M, Arias AM, Maniatis T (1990) Expression of a MyoD family member prefigures muscle pattern in Drosophila embryos. Genes Dev 4: 2086–2097. 2176634 10.1101/gad.4.12a.2086

[19] Meedel TH, Farmer SC, Lee JJ (1997) The single MyoD family gene of Ciona intestinalis encodes two differentially expressed proteins: implications for the evolution of chordate muscle gene regulation. Development 124: 1711–1721. 9165119 10.1242/dev.124.9.1711

[20] Araki I, Terazawa K, Satoh N (1996) Duplication of an amphioxus myogenic bHLH gene is independent of vertebrate myogenic bHLH gene duplication. Gene 171: 231–236. 8666278 10.1016/0378-1119(96)00174-6

[21] Hedges SB (2002) The origin and evolution of model organisms. Nat Rev Genet 3: 838–849. 12415314 10.1038/nrg929

[22] Schubert M, Meulemans D, Bronner-Fraser M, Holland LZ, Holland ND (2003) Differential mesodermal expression of two amphioxus MyoD family members (AmphiMRF1 and AmphiMRF2). Gene Expr Patterns 3: 199–202. 12711549 10.1016/s1567-133x(02)00099-6

[23] Urano A, Suzuki MM, Zhang P, Satoh N, Satoh G (2003) Expression of muscle-related genes and two MyoD genes during amphioxus notochord development. Evol Dev 5: 447–458. 12950624 10.1046/j.1525-142x.2003.03051.x

[24] Meedel TH, Lee JJ, Whittaker JR (2002) Muscle development and lineage-specific expression of CiMDF, the MyoD-family gene of Ciona intestinalis. Dev Biol 241: 238–246. 11784108 10.1006/dbio.2001.0511

[25] Holland PW, Garcia-Fernandez J, Williams NA, Sidow A (1994) Gene duplications and the origins of vertebrate development. Dev Suppl: 125–133. 7579513

[26] Marchler-Bauer A, Lu S, Anderson JB, Chitsaz F, Derbyshire MK, et al (2011) CDD: a Conserved Domain Database for the functional annotation of proteins. Nucleic Acids Res 39: D225–229. 21109532 10.1093/nar/gkq1189 PMC3013737

[27] Yang Z (2007) PAML 4: phylogenetic analysis by maximum likelihood. Mol Biol Evol 24: 1586–1591. 17483113 10.1093/molbev/msm088

[28] Yang Z (1998) Likelihood ratio tests for detecting positive selection and application to primate lysozyme evolution. Mol Biol Evol 15: 568–573. 9580986 10.1093/oxfordjournals.molbev.a025957

[29] Yang Z, Nielsen R (2000) Estimating synonymous and nonsynonymous substitution rates under realistic evolutionary models. Mol Biol Evol 17: 32–43. 10666704 10.1093/oxfordjournals.molbev.a026236

[30] Yang Z, Nielsen R (2002) Codon-substitution models for detecting molecular adaptation at individual sites along specific lineages. Mol Biol Evol 19: 908–917. 12032247 10.1093/oxfordjournals.molbev.a004148

[31] Gu X, Vander Velden K (2002) DIVERGE: phylogeny-based analysis for functional-structural divergence of a protein family. Bioinformatics 18: 500–501. 11934757 10.1093/bioinformatics/18.3.500

[32] Swanson WJ, Yang Z, Wolfner MF, Aquadro CF (2001) Positive Darwinian selection drives the evolution of several female reproductive proteins in mammals. Proc Natl Acad Sci U S A 98: 2509–2514. 11226269 10.1073/pnas.051605998 PMC30168

[33] Clark NL, Swanson WJ (2005) Pervasive adaptive evolution in primate seminal proteins. PLoS Genet 1: e35. 16170411 10.1371/journal.pgen.0010035 PMC1201370

[34] Zhao X, Mo D, Li A, Gong W, Xiao S, et al (2011) Comparative analyses by sequencing of transcriptomes during skeletal muscle development between pig breeds differing in muscle growth rate and fatness. PLoS One 6: e19774. 21637832 10.1371/journal.pone.0019774 PMC3102668

[35] Meedel TH, Chang P, Yasuo H (2007) Muscle development in Ciona intestinalis requires the b-HLH myogenic regulatory factor gene Ci-MRF. Dev Biol 302: 333–344. 17055476 10.1016/j.ydbio.2006.09.043 PMC1797879

[36] Yokoyama S, Asahara H (2011) The myogenic transcriptional network. Cell Mol Life Sci 68: 1843–1849. 21318263 10.1007/s00018-011-0629-2 PMC3092062

[37] Mok GF, Sweetman D (2011) Many routes to the same destination: lessons from skeletal muscle development. Reproduction 141: 301–312. 21183656 10.1530/REP-10-0394

[38] Koumans JTM, Akster HA (1995) Myogenic cells in development and growth of fish. Comparative Biochemistry and Physiology Part A: Physiology 110: 3–20.

[39] Rescan PY (2001) Regulation and functions of myogenic regulatory factors in lower vertebrates. Comp Biochem Physiol B Biochem Mol Biol 130: 1–12. 11470439 10.1016/s1096-4959(01)00412-2

[40] Della Gaspera B, Armand AS, Sequeira I, Chesneau A, Mazabraud A, et al (2012) Myogenic waves and myogenic programs during Xenopus embryonic myogenesis. Dev Dyn 241: 995–1007. 22434732 10.1002/dvdy.23780

[41] Innocenzi A, Latella L, Messina G, Simonatto M, Marullo F, et al (2011) An evolutionarily acquired genotoxic response discriminates MyoD from Myf5, and differentially regulates hypaxial and epaxial myogenesis. EMBO Rep 12: 164–171. 21212806 10.1038/embor.2010.195 PMC3049428

[42] Tamura K, Peterson D, Peterson N, Stecher G, Nei M, et al (2011) MEGA5: molecular evolutionary genetics analysis using maximum likelihood, evolutionary distance, and maximum parsimony methods. Mol Biol Evol 28: 2731–2739. 21546353 10.1093/molbev/msr121 PMC3203626

[43] Yang Z, Nielsen R (1998) Synonymous and nonsynonymous rate variation in nuclear genes of mammals. J Mol Evol 46: 409–418. 9541535 10.1007/pl00006320

[44] Kelley LA, Sternberg MJ (2009) Protein structure prediction on the Web: a case study using the Phyre server. Nat Protoc 4: 363–371. 19247286 10.1038/nprot.2009.2

[45] Sayle RA, Milner-White EJ (1995) RASMOL: biomolecular graphics for all. Trends Biochem Sci 20: 374. 7482707 10.1016/s0968-0004(00)89080-5

[46] Goodsell DS (2005) Representing structural information with RasMol. Curr Protoc Bioinformatics Chapter 5: Unit 5 4. 10.1002/0471250953.bi0504s11 18428750

[47] Gu X (1999) Statistical methods for testing functional divergence after gene duplication. Mol Biol Evol 16: 1664–1674. 10605109 10.1093/oxfordjournals.molbev.a026080

[48] Gu X (2001) Maximum-likelihood approach for gene family evolution under functional divergence. Mol Biol Evol 18: 453–464. 11264396 10.1093/oxfordjournals.molbev.a003824

