# Correction: Patterns of Positive Selection of the Myogenic Regulatory Factor Gene Family in Vertebrates

**DOI:** 10.1371/journal.pone.0101726

**Published:** 2014-06-25

**Authors:** The PLOS ONE Staff

There are errors in the Funding section. The correct funding information is as follows: This work was supported by the National Basic Research Program of China (2012CB114600), National Natural Science Foundation of China (31240037, 31301951) and Postdoctoral Science Foundation of China (2013M531638). The funders had no role in study design, data collection and analysis, decision to publish, or preparation of the manuscript.
